# A Case Report on Core Muscles Training for Knee Osteoarthritis Through Core Muscles Activations and Gait Analysis

**DOI:** 10.7759/cureus.33918

**Published:** 2023-01-18

**Authors:** D. Maryama Ag Daud, Shye Nee Liau, Suhaini Sudi, Malehah Mohd Noh, Nyein Yin Khin

**Affiliations:** 1 Healthy Through Exercise and Active Living Research Unit, Faculty of Medicine and Health Sciences, Universiti Malaysia Sabah, Kota Kinabalu, MYS; 2 Biomedical Sciences, Faculty of Medicine and Health Sciences, Universiti Malaysia Sabah, Kota Kinabalu, MYS; 3 Internal Medicine, Faculty of Medicine and Health Sciences, Universiti Malaysia Sabah, Kota Kinabalu, MYS; 4 Rehabilitation Medicine, Faculty of Medicine and Health Sciences, Universiti Malaysia Sabah, Kota Kinabalu, MYS

**Keywords:** electromyogram, walking gait, knee pain, womac, resistance training, core muscles, knee osteoarthritis

## Abstract

Knee osteoarthritis (OA) is a chronic joint disease that can affect all ages, but it is more common in the elderly. Pharmacological and non-pharmacological treatments have been invented evolutionarily over the years to halt this disease. Exercise is one of the first-line treatments for knee OA as well as for prevention. This case study features a 47-year-old man who has grade IV bilateral knee OA and has never had any surgery and takes fish oil daily as a supplement. His walking pattern was significantly impacted by the chronic knee discomfort he had in both legs. Thus, the walking gait of this patient was analyzed together with core muscle activation before and after two weeks of core resistance exercise intervention. The knee pain score was assessed using the Western Ontario and McMaster Universities Index (WOMAC). The outcomes of this research depict that core resistance training has the potential to be used as an alternative, non-surgical and non-pharmacological treatment for a patient with knee OA.

## Introduction

Osteoarthritis of the knee joint (OA) is the most common joint disease [1] and is commonly associated with pain and disability [2]. OA knee is caused by the degeneration of the cartilage tissue between the bones that can affect walking gait. Knee OA patients alter their walking gait patterns by shifting the body weight to different joints to compensate for joint pain and instability [3]. For some patients, issues with pelvis stabilization during gait that leads to knee bending lameness are made worse by muscular wasting and weakness that spread proximally to the thigh muscles [3,4].

As we know, core muscles play a significant role in walking ambulation as it consists of static and dynamic at the zone that acts as a base to provide mobility movements. Core muscles consist of several groups of muscles, such as the gluteus, hip, abdominal, and spine, which cover the abdominal trunk to the lower torso that act for in-body stabilization, postural control, and force production in daily life routines [5]. Instead, when core muscles get weaker, some asymmetries can be detected for the gait outcomes [6], limb loading [7], and muscle activation [8]. Weak core musculature or trunk control to support body weight increases knee pain intensity that impairs walking among patients suffering from OA on the knee joint [9]. Are people who suffered from OA in their knees going to benefit from core training to improve their walking gait? Thus, this study looked at changes in core muscle recruitment during walking after a two-week core training program to better understand the role of core muscles during walking in patients with OA knee.

Here, we report a case of preliminary research to study the potential of core muscle training on alterations of core muscle activation, gait analysis, and knee pain among knee OA patients.

## Case presentation

A 47-year-old businessman with 178cm and 77.3kg was referred to our clinic with chronic knee pain in both legs. From his medical history, he never had any surgery and took fish oil soft gel daily as a supplement. His job does not require a lot of standing or heeled shoes and does not cause mental stress or tension. In his spare time, he enjoys gardening. However, he claimed that his chronic knee discomfort from OA was making it difficult for him to walk. He is otherwise healthy, with no known chronic diseases, infections, immune disorders, or such.

He had sought medical attention for his knee pain by visiting his primary care provider. A rehab physician from Polyclinic Kingfisher, University Malaysia Sabah (UMS) diagnosed him with grade IV OA in both his left and right knees. He was prescribed a training program with significant improvement. The patient underwent physical activity readiness screening and was cleared to undergo exercise training. Therefore, six sessions of two weeks of core muscle exercise training were prescribed according to his physical ability, especially with a limited range of motion on the knee joint. The training program consisted of various modified core exercises such as static cycling, rowing, leg raise, trunk curl, abs curl, trunk extension, and modified Russian twist. The two weeks training program, with 1 hour 30 minutes per session with a training frequency of three sessions per week (Table 1).

**Table 1:** Summary of the core muscles training protocol # Each exercise was done with three sets of 12 to 15 repetitions and 45-sec to 60-sec rest interval between sets. * Each exercise was done with intensity set 40% to 70% predicted 1RM, depending on the patient's progression.

Session #	Exercise	Targeted core muscles
1	SET 1 Back extension (machine)* Single leg lift# Abs crunch (machine)* Static cycling for 5 minutes (machine)	SET 1 Erector spinae & Gluteus maximus Transverse abdominis & Hamstrings Rectus abdominis Quadriceps
	SET 2 Curl ups# Lying single straight leg raise# Abs crunch (machine)* Rowing for 5 minutes (machine)	SET 2 Rectus abdominis & Obliques Iliopsoas, Rectus abdominis & Obliques Rectus abdominis Quadriceps
2	SET 1 Seated knee tucks# Alternating heel touch# Exercise ball Russian twist# Alternating knee to chest#	SET 1 Abductor longus & Iliopsoas Obliques & Transverse abdominis Rectus abdominis & Obliques Iliopsoas & Quadriceps
	SET 2 Bent leg raises# Bench flutter kicks# Ab bicycles# Ball V-ups#	SET 2 Rectus abdominis Glutes & Hamstrings Rectus Abdominis & Obliques Rectus Abdominis & Transverse Abdominis
3	SET 1 Clamshells# Curl-ups# Seated alphabet leg writing (A-Z)# Dead bugs#	SET 1 Glutes & Iliopsoas Rectus abdominis & Obliques Quadriceps & Hamstrings Rectus abdominis
	SET 2 Rowing for 8 minutes (machine) Abs crunch (machine)* Flutter kicks#	SET 2 Quadriceps Rectus abdominis & Obliques Rectus abdominis
4	SET 1 Hip bridge# Lateral leg raise (lying down)# Raise leg crunches# Top single-leg circles#	SET 1 Glutes, Iliopsoas & Hamstrings Glutes & Iliopsoas Rectus abdominis & Transverse abdominis Rectus abdominis
	SET 2 Prone straight leg raise# Single leg lift# Oblique-twist triceps push-up# Dancing bugs#	SET 2 Glutes & Iliopsoas Transverse abdominis & Hamstrings Gluteus medius, Obliques & Triceps Rectus abdominis, Transverse abdominis & Obliques
5	SET 1 Superman Rowing for 10 minutes (machine) Abs crunch (machine)* Alternating heel touch#	SET 1 Hamstrings, Erector spinae & Glutes Quadriceps Rectus abdominis Obliques & Transverse abdominis
	SET 2 Shin touches# Seated single-leg circles# Leg lift knee in# Inner thigh lifts#	SET 2 Rectus abdominis & Transverse abdominis Glutes Quadriceps & Hip abductors Adductor longus
6	SET 1 Prone straight leg raise# Clamshells# Reaches# Side leg circles#	SET 1 Glutes & Iliopsoas Glutes & Iliopsoas Glutes & Iliopsoas Gluteus maximus, Hip flexors, Abductors & Adductors
	SET 2 Hip bridge with resistance band# Superman# Sit-up# Seated alphabet leg writing (A-Z)#	SET 2 Glutes, Hip flexors & Hamstrings Hamstrings, Erector spinae & Glutes Rectus abdominis & Transverse abdominis Quadriceps & Hamstrings

The progress of his knee pain was then evaluated as the sessions progressed by completing the WOMAC pain subscale questionnaire at the end of every training session. In total, six WOMAC pain subscales were recorded for six sessions of core muscle training on the patient for two weeks (Table 2). The mean score from the subscales was reduced for every session of core resistance training.

**Table 2:** WOMAC pain score

Session	Total WOMAC Score
1	42
2	40
3	34
4	28
5	25
6	20

Before and immediately after the training program, kinetic analysis of gait (center of pressure and spatial gait parameters) was assessed using the Zebris FDM-T Treadmill [10], and selected core muscle activation during walking gait assessment was analyzed. As for walking protocol, the patient is required to walk on the flat treadmill with zero gradients for one minute at his preferred walking speed (1.8km/h). The electrodes were then placed on the selected core muscles (Lumbar Erector Spinae, ES; and Rectus Abdominis, RA) and two lower limb muscles (Vastus Lateralis, VL; and Rectus Femoris RF), which are vital in assisting the lower part of body mobilities such as walking, in this case [11].

Based on the walking gait cycle, the center of pressure parameters (COP) and gait spatial were assessed: force pressure distribution, length of gait line, anterior/posterior position, cadence, stride length, and foot rotation.

For COP, Table 3 displays the COP parameters of the gait analysis by the patient. The length of the gait line for the patient increased after two weeks of core muscle training for both the left and right foot, from only 212mm to 222mm and 214mm to 221mm, respectively (Table 3). Therefore, the deviation in the gait line length decreased to almost zero percent or became symmetry with two weeks of core muscles training intervention.

**Table 3:** Changes in spatiotemporal gait after two weeks of core muscles training

	Length of Gait Line, mm			Anterior/Posterior mm
	LT	RT	% difference	
Pre	212 ± 8	214 ± 5	1.3	147 ± 4
Post	222 ± 15	221 ± 6	-0.2	166 ± 5
Changes (%)	4.70%	3.30%	Pressure distributes to the anterior of the foot	

**Figure 1:**
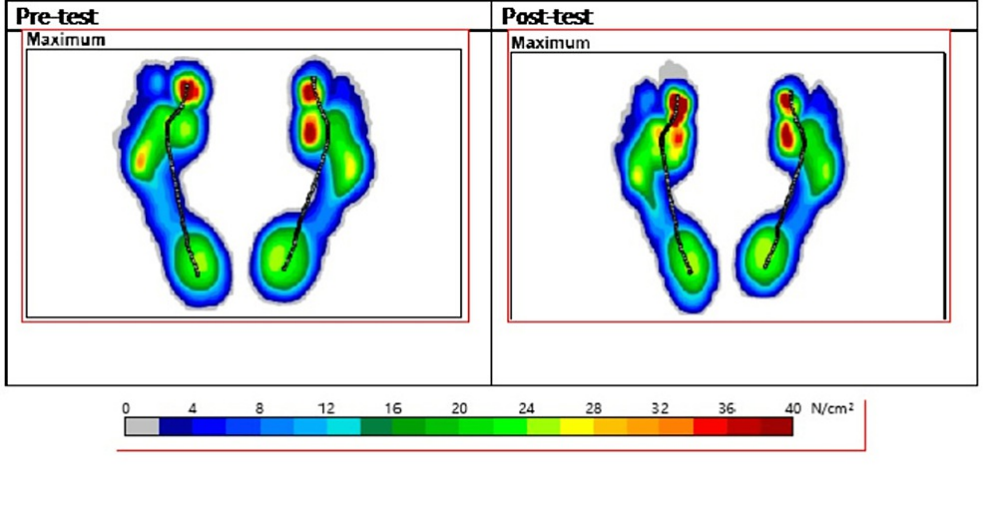
Feet pressure distribution during walking on the treadmill.

Figure 1 shows that the post-intervention did not significantly alter the maximum force and pressure distributions for the preferred leg (right). Instead, maximum force and pressure have distributed not only on the thumb but also over the balls of the left foot. This finding is correlated with the anterior/posterior position outcomes that showed that the foot position has increased from 147mm during the pre-test to 166mm post-test (Table 3). Increased value for foot position is represented by the forward shift of the centre of pressure intersection point to the anterior part of the foot, which is the thumb and the ball.

Table 4 demonstrates the two weeks of core muscles training intervention affects the spatial gait parameters, which has changed the left foot rotation from only 10.6 ° to 11.9 °, enhancing the normal foot rotation angle, 18.5 ± 8.15 ° [12]. Even though the right foot rotation remains unchanged as it is in the normal range of degrees, the rise of step length for the left and the right foot is also recorded, from 44cm to 45cm and 48cm to 52cm, respectively, in accordance with the cadence, from 88 step/min to 95 step/min accounts for eight percent increase (Table 4).

**Table 4:** Changes in the gait spatial parameters after two-week of core muscles training intervention

	Foot Rotation ( ° )		Step Length cm			Cadence Step/min
	LT	RT	LT	RT	% dev.	
Pre	10.6 ± 1.7	18.1 ± 1.8	44 ± 3	4 ± 4	8.9	88 ± 4
Post	11.9 ± 1.5	18.0 ± 1.8	45 ± 2	5 ± 2	15.8	95 ± 2
Changes (%)	Increase	No change	2.30%	8.30%	Increase	8%

To further confirm whether changes in his walking gait are due to the increase in core muscle recruitment, core muscle activation during walking was analyzed. A portable EMG system (Myomonitor, Delsys Inc., Boston, MA, USA) sEMG was used to analyze the muscles' activation while performing walking gait analysis. Muscle activation level was measured using the root mean square (RMS) Value of the average power of the EMG signal for a one-minute duration during gait analysis. The magnitude of differences in RMS values between pre and post were quantified and are reported as the percentage differences.

EMG electrodes (DE-2.3, 5-mm single differential surface EMG sensor with two 1-mm Ag contacts 10 mm apart) were positioned longitudinally on the belly of each muscle with respect to the underlying muscle fibers in accordance with standard recommendations to minimize cross-talk and geometrical artifacts.

**Figure 2:**
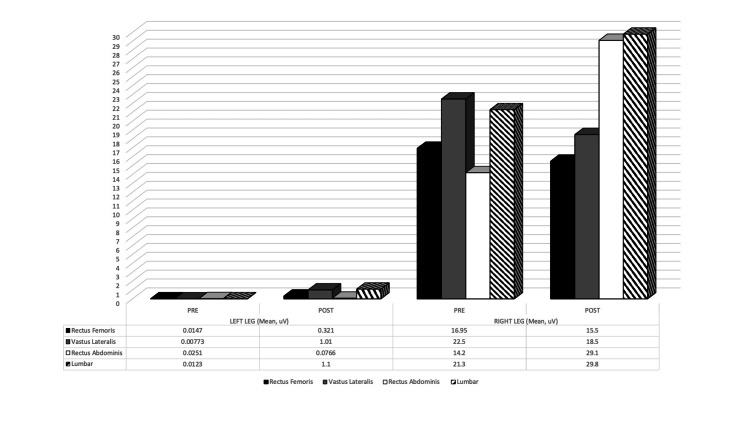
The mean amplitude EMG signals of core muscles for both left and right legs during 1 minute walking gait analysis

In this case study, the mean amplitude of EMG signals of the selected muscles (Rectus Abdominis, Lumbar, Rectus Femoris, and Vastus Lateralis) while 1-minute walking on the treadmill was analyzed. As expected, Figure 2 shows muscle activation of the Rectus Femoris, Vastus Lateralis, Rectus Abdominis, and Lumbar for the left leg increased remarkably after the core muscles training regimen. This means more recruitment of the respective core muscles after the core exercise intervention. Furthermore, this figure also depicts the muscle activation of the right Rectus Abdominis and right Lumbar during the post-test increased but not as much as the left leg. More rectus abdominis and lumbar muscles were recruited after the exercise intervention. However, the right Rectus Femoris and Vastus Lateralis during the post-test are lower than in the pre-test. This indicates less muscle recruitment for Rectus Femoris and Vastus Lateralis after the exercise intervention.

## Discussion

According to the gait analysis outcomes, the distribution of weight and pressure concentrated significantly on the thumb, followed by the balls and the heel of the foot, and exercise intervention did not significantly alter these distributions. Instead, tension is still built up at the toes. This odd finding is correlated with the significant rise of the anterior shift of the center of pressure for the intersection point [13] towards the anterior part of the foot. This is probably due to the concentration of the core muscles training regimen instead of intervening in steps exercise. Furthermore, the core muscles training program involved static cycling, which requires a good cycling posture to produce optimal trunk flexion. However, a good cycling posture is sometimes a very subjective matter that must depend on the experience of the patient [14] in this case study. Over-trunk flexion will create potential risks of strain on the skeleton and muscles [14]. In this case, the concentration of an unpractical weight or pressure distribution is still detected significantly on the anterior part of the feet, which can expose the high risk of injuries on the metatarsal part.

On the other hand, the two weeks of core muscle training intervention did help the patient in improving the gait line length and nearly becoming symmetry (from 1.3% to 0.2%); increased the left foot rotation angle (from 10.6% to 11.9%) to normal range (±18.0%) [15]. Although the right foot rotation angle remained constant, the rise of the stride length and velocity with the same speed is consistent with the previous research outcomes that analyzed the gait analysis after the core stabilization regimen intervention [16]. Trunk coordination could influence the gait parameters, as trunk coordination can change the walking velocity of a person [16].

During the post-test, the muscle activation of the right rectus abdominis and right lumbar increased but not as much as the left leg (Figure 2). This indicates that more rectus abdominis and lumbar muscles were recruited after the exercise intervention. However, the right rectus femoris and vastus lateralis during the post-test are lower than in the pre-test. Likely, less muscle recruitment for the rectus femoris and vastus lateralis after the core muscles training intervention due to the right rectus abdominis and right lumbar, which are categorized as part of the big muscles in the core, are strengthened, less tension or pressure is exerted on the small muscles in right quads (rectus femoris and vastus lateralis). Furthermore, this feasible finding is supported by a few research studies [17,18].

During the pre-test, most muscle recruitment was focused on the lower extremities (vastus lateralis and rectus femoris). In contrast, the major muscles (rectus abdominis and lumbar) mean activation was less, indicating that the patient did not recruit lots of abdominal and back muscles while performing a walking test on the treadmill. In addition, high muscle contraction of the small muscles can be linked to knee OA severity, which is presumed to also relate to higher muscle force to compensate the joint instability [19]. This research has shown that strong abdominal and back muscles can actively recruit these muscles during even mild daily activities such as walking and putting on shoes. Inactive use of the abdominal and back muscles can induce more tension on the smaller muscles, such as, in this case, the rectus femoris and vastus lateralis. Albeit the big muscles assist more in performing daily activities that can take up less pressure and achieve the optimum performance. For instance, a sprinter generates power from the big muscle groups, such as ‘the glute muscles’ instead of the small muscle, like the vastus lateralis.

Activating the major muscle is essential to not put tension or pressure on the small muscle that can lead to injury in the athletes [20]. In this case, our knee OA patient, in turn, can cause pain and discomfort in the knee. Specifically, the recruitment of only a single muscle group can exert excessive loads on the said muscles and lack activation of the more prominent featured muscles that are more than ready to serve its purpose. Based on the post-test outcomes, the two-week supinated and seated core muscle training regimen proves to be used by transferring the muscle recruitment to the big muscles. This can be seen in the right knee, especially where less mean muscle activation of the small muscles occurred instead of slightly muscular activation of the big muscles that used to be less activated during the pre-test.

## Conclusions

Knee osteoarthritis (OA) causes reduced mobility, i.e., gait walking. Patients who experience discomfort or joint instability change their walking techniques by transferring loads to joints like the hip and ankle and changing patterns of muscle activation. This study found that core muscles were activated or recruited while walking. After only two weeks, core exercise was shown to effectively reduce knee pain and improve walking gait in this chronic knee OA patient. This study discovered that two weeks of core training helped improve walking gait by enhancing core muscle recruitment, specifically the Rectus Abdominus and Lumbar. As a result, core training can be inferred to be effective in the physical rehabilitation of knee OA patients, particularly in terms of improving their gait. These findings raise the topic of the optimal load and exercise selection for strengthening core muscles in knee OA patients. Various modified core exercises that include low-intensity, relaxed activities, and repetition that can be adjusted based on the patient's physical ability can enhance physical ability and restore muscle strength in knee OA patients. A particular training regimen to enhance gait will need to be developed in the future.
